# Objectively Measured Physical Activity and Body Mass Index in Preschool Children

**DOI:** 10.1155/2010/479439

**Published:** 2010-07-11

**Authors:** Susana Maria Coelho Guimarães Vale, Rute Marina Roberto Santos, Luísa Maria da Cruz Soares-Miranda, Carla Marisa Maia Moreira, Jonatan R. Ruiz, Jorge Augusto Silva Mota

**Affiliations:** ^1^Research Centre in Physical Activity, Health and Leisure, Faculty of Sport, Porto University, 4200-450 Porto, Portugal; ^2^Department of Biosciences and Nutrition, Unit for Preventive Nutrition, Karolinska Institute, 14183 Stockholm, Sweden

## Abstract

*Aim*. To examine the association between objectively measured physical activity (PA) and body mass index (BMI) in preschool children. *Methods*. The study comprised 281 children (55.9% boys) aged from 4 to 6 years. PA was measured by accelerometer. Children were categorized as non-overweight (NOW) and overweight/obese (OW) according to the sex-adjusted BMI z-score (<1 and ≥1, resp.). *Results*. Total and moderate intensity PA were not associated with BMI. We observed that a higher proportion of OW children were classified as low-vigorous PA compared to their NOW peers (43.9 versus 32.1%, resp., *P* > .05). Logistic regression analysis showed that children with low-vigorous PA had higher odds ratio (OR) to be classified as OW compared to those with high-vigorous PA (OR = 4.4; 95% CI: 1.4–13.4; *P* = .008) after adjusting for BMI at first and second years of life and other potential confounders. *Conclusion*. The data suggests that vigorous PA may play a key role in the obesity development already at pre-school age.

## 1. Introduction

The prevalence of childhood obesity has been rising during the past decades in many parts of the world [1]. In Portugal, there is a high prevalence of overweight and obese children [2] and adolescents [3]. This picture is particularly alarming owing to the increasing risk of developing cardiovascular diseases in overweight and obese individuals [4, 5]. Over the long term, childhood/adolescence overweight is strongly associated with adult obesity [6, 7]. Therefore, it is of clinical and public health importance to examine the risk trends in order to develop effective preventive strategies targeting those at risk start as early as possible. 

Human obesity is a multifactorial disorder where both genes [8] and lifestyle factors, including diet and physical activity [9] are important contributors. Both maternal and paternal body mass index (BMI) has also a strong influence on offspring's risk of obesity [10, 11]. Other determinants of childhood obesity include birth weight and weight gain that occur during the first years of life [12–14]. 

It has been suggested that obesity during the pre-school years is associated with other clinical factors easily assessed at birth [15]. For instance, it was found an association between birth weight and the risk of being obese in children at the age of 4, 8, 10, and 12 years [16]. 

Besides the previously mentioned factors, there exist other potentially modifiable factors that increase the risk of overweight in childhood and adolescence. These include: (i) intrauterine life: excessive gestational weight gain [17, 18], and maternal smoking during pregnancy [13, 19, 20]; (ii) infancy and pre-school period: reduced breastfeeding duration [21], excessive weight gain in the first 2 years of life [12, 22], excessive television [23–25], short sleep duration [12, 26, 27], and low levels of physical activity (PA) [28–30].

Studies examining the associations between PA and body fat in young children are scarce [12, 28, 30], and to the best of our knowledge, few studies have estimated the associations between objectively measured PA and BMI in preschoolers [28, 30]. Furthermore, there is no information available in Portuguese population.

The purpose of this study was to analyze the association between objectively measured PA and BMI in Portuguese preschoolers.

## 2. Methods

### 2.1. Participants and Data Collection

This is a cross-sectional study carried out in Portuguese (metropolitan area of Porto) kindergartens enrolled in the Preschool Physical Activity, Body Composition and Lifestyle Study (PRESTYLE). A total of 281 healthy pre-school children (55.9% boys) aged 4–6 years with complete information on the variables of interest were included in the study. Data collection took place between April 2009 and November 2009.

Informed written consent was obtained from parents and school supervisors. Study procedures were approved by the Portuguese Foundation for Science and Technology and by the Scientific Board of Physical Activity and Health PhD program.

### 2.2. Anthropometric Measures

Body weight and height were measured by standard anthropometric methods. Body weight was measured to the nearest 0.10 kg, with participants lightly dressed (underwear and tee-shirt) using a portable digital beam scale (Tanita Inner Scan BC 532). Body height was measured to the nearest millimetre in bare or stocking feet with children standing upright against a Holtain portable stadiometer (Tanita). The measurements were repeated twice and the average was recorded. BMI was calculated as body mass (kg) divided by height (m) squared. Children were classified as either non-overweight (NOW) or overweight (OW) according to the sex-adjusted BMI z-score (<1 SD and ≥1 SD, respectively). Children were evaluated during school day by trained teachers.

### 2.3. Physical Activity

PA was measured using Actigraph accelerometers, model GTM1 (Pensacola, FL 32502. USA). This is a small, lightweight, uniaxial device. This accelerometer produces “raw” output in activity counts per minute (cpm), which gives information about the total amount of PA [31]. The accelerometer output can also be interpreted using specific cut points, which describes different PA intensities PA. Data reduction, cleaning, and analyses of accelerometer data were performed as described elsewhere [32, 33]. Data were analysed using specific paediatric cut points, which have been validated for young children: ≥1100 and ≤1680 cpm for low PA [34], >1680 cpm for moderate PA, and >3360 cpm for vigorous PA (VPA) [35]. In this study, the epoch duration was set to 5 seconds, which seems to be more accurate and suitable concerning the spontaneous and intermittent activities of the young children [36]. 

A minimum of 10 hours per day was considered as valid data for the analysis. Parents were instructed to place the accelerometer on the child right after waking up and remove it before going to sleep. The accelerometer was adjusted at the child's right hip by an elastic waist belt under clothing (own cloth and school coat). A data sheet was given to the children's teachers, who were instructed to record the time when the child arrived and left the school. Activities were not prescribed or directed by the teachers or researchers. All children participated in normal activities with their classmates. 

All PA intensity levels were defined by sex- and age-specific tertiles. Children belonging to the first, second, and third tertiles were defined as low, middle and high PA levels, respectively.

### 2.4. Potential Confounders

#### 2.4.1. Pre- and Postnatal and Lifestyle Factors

Mothers reported information regarding gestational weight gain, maternal smoking during pregnancy, birth weight as well as body weight and height during their offspring's first and second year of life. Gestational weight gain was categorized according to Institute of Medicine [37] as below, optimal, and above gestational weight gain, while maternal smoking during pregnancy was categorized as YES or NO.

Mothers also reported the amount of screen time (watching television and/or playing videogames) the child spends daily as well as the sleeping time for both week days and weekends. Screen time questions were analyzed as continuous variables (converted to minutes) and also evaluated as a dichotomous variable based on young children recommendation [38]. Then, children were classified as those who accomplished guidelines (watching <2 hours/day) and those who did not (watching ≥2 hours/day).

#### 2.4.2. Mother Information

Mothers reported their body weight and height, and we calculated BMI. Mothers were categorized as normal weight (18.5 kg/m^2^ ≥ BMI <25 kg/m^2^); overweight (25 kg/m^2^ ≥ BMI <30 kg/m^2^), and obese (BMI ≥30 kg/m^2^) [39].

Socioeconomic status (SES) was defined as the mother's educational level and occupation [40]. The SES was defined based upon Portuguese Educational system a 9 years' education or less subsecondary level (scored as 1), 10–12 years' education, secondary level (scored as 2) and higher education (scored as 3). Levels 1, 2, and 3 were considered as low, middle, and high SES [41].

### 2.5. Statistical Analysis

Means and standard deviations were calculated to describe children's characteristics by weight status (i.e., NOW and OW). 

Comparisons between weight status and PA patterns were conducted with *t*-test for continuous variables and chi-square test for categorical variables.

Following bivariates correlation analysis we conducted logistic regression to examine the association between weight status and all other variables (physical activity patterns, gestational weight gain, smoking during pregnancy, BMI first year of life, BMI second year of life, daily screen time, daily sleep time). 

A stepwise logistic regression analysis was performed to examine the association between PA and weight status, adjusted for all variables independently associated with weight status.

Statistical analysis was performed using the SPSS 17.0 software (SPSS Inc., Chicago, IL, USA). The level of significance was set at *P* ≤ .05.

## 3. Results

Table 1 shows descriptive statistics of preschoolers and parents by overweight status. The prevalence of overweight was 14.6%. Overweight (OW) children were heavier, taller, and had higher BMI than their NOW counterparts (*P* ≤ .05). We observed no statistical significant differences between weight status categories in minutes of total, MPA and VPA. However, the data showed that a proportion of OW children (43.9%) were classified low VPA compared to NOW children (32.1%) (*P* > .05).

Logistic regression analysis showed that children with low vigorous PA had higher odds ratio (OR) to be classified as OW compared to those with high vigorous PA (OR = 4.4; 95% CI: 1.4–13.4; *P* = .008) after adjusting for BMI at first and second years of life and other potential confounders (Table 2). 

## 4. Discussion

This study examined the association of different PA intensity levels with weight status of Portuguese preschoolers after adjusting for several potential confounding factors. This is an important and relevant topic since, to the best of our knowledge, little is known about how PA intensity is associated with obesity in pre-school children. Our data showed that differences in levels of VPA were associated with weight status in children as young as 4 to 6 years. This is worthy to notice because our data suggest that the VPA influenced the change in BMI from those earlier ages. Despite that, no statistical significant differences were found for levels of total and moderate PA. 

Our findings concur with other studies showing that low levels of VPA were associated with body fatness during the adiposity rebound period [30]. Further, they also agree with studies in children and adolescents showing that only VPA (but not lower intensity levels) was associated with body fat [42]. Additionally, it was shown that within intervention groups, those who participated regularly and maintained the highest heart rates during PA sessions showed the greatest decreases in body fat and the greatest increases in bone density [43, 44]. On the other hand, adolescents who engaged in relatively large amounts of free-living vigorous PA were likely to be relatively fit and lean. [45]. These findings are worth commenting in terms of both PA interventions and public health policies.

The large standard deviations found in our study suggest a wide individual variations and highlight the importance of the participants' intraindividual variability in PA behaviour. Therefore, variation in PA levels may be particularly important in preschool children with regard to weight status. While there is a need to better understand the factors that influence PA in preschoolers and to learn how to help them to become more active, our study shows that PA promotion and interventions should focus on the more intense PA activities. Children have a natural tendency towards movement, there is information suggesting a decline of discretionary time on children's daily life [29] and, thus, the time allocated to spontaneous PA, which, in turn, tend to be highly active [46] it is reduced and several sedentary behaviors such as TV viewing, video games, and other activities involving many hours standing took the lead on children's daily behaviour [12, 24]. Therefore, promotion of organized PA programmes such as physical education at schools and organized sports activities [29] that usually request more intense activities must be taken into account when PA promotion strategies are being developed.

Some limitations of this study should be recognized. First, the study included pre-school children from one metropolitan area, which made it difficult to generalize these findings. Secondly, it is not possible to inferr causal relationships between pre-school PA level and overweight status with such a cross-sectional study design. Nevertheless, this study focuses on the assessment of PA levels in a pre-school sample using an objective measure, which enhances the confidence of our findings owing to the fact that accelerometers provide more valid PA assessment in children [35].

## 5. Conclusion

Our data suggests that VPA may play a key role in the obesity development already at pre-school age.

## Figures and Tables

**Table 1:** Descriptive statistics of study participants.

	All Group	N-OW	OW	*P*
	*N* = 281	*N* = 240	*N* = 41	
Age (years)	5.03 ± 0.81	5.01 ± 0.82	5.14 ± 0.72	.264
Weight (Kg)	21.11 ± 4.42	20.12 ± 3.14	27.92 ± 5.73	<**.001**
Height (m)	1.11 ± 0.08	1.10 ± 0.08	1.14 ± 0.08	**.005**
BMI (Kg /m^2^)	17.03 ± 2.12	16.43 ± 1.29	21.11 ± 2.20	<**.001**
BMI z-score		−0.28 ± 0.61	1.92 ± 1.04	<**.001**
TPA (minutes)	134 ± 35	134 ± 36	133 ± 29	.863
MPA (minutes)	58 ± 14	58 ± 14	58 ± 13	.882
VPA (minutes)	38 ± 14	38 ± 14	35 ± 12	.293
Physical Activity Patterns (%)				
TPA				
Low Activity	32.4	33.3	26.8	
Middle Activity	34.9	32.9	46.3	.249
High Activity	32.7	33.8	26.8	
MPA				
Low MPA	32.4	33.3	26.8	
Middle MPA	35.2	33.3	46.3	.273
High MPA	32.4	33.3	26.8	
VPA				
Low VIG	33.8	32.1	43.9	
Middle VIG	33.5	32.5	39.0	.064
High VIG	32.7	35.4	17.1	

TPA: Total Physical Activity; MPA: Moderate Physical Activity; VPA: Vigorous Physical Activity.

**Table 2:** Univariable and multivariable logistic regressions.

	Univariable Effects (odds ratio (95% CI))	*P* value		Multivariable Stepwise effects*(odds ratio (95% CI))	*P* value
TPA (Low TERTILE)	1.7 (0.8–3.9)	>.05			
TPA (Middle TERTILE)	1.0 (0.4–2.5)	>.05			
TPA (High TERTIL)—REF					
MPA (Low TERTILE)	1.7 (0.8–3.9)	>.05			
MPA (Middle TERTILE)	1.0 (0.4–2.4)	>.05			
MPA (High TERTIL)—REF					
*VPA (Low TERTILE)*	**2.8 (1.1**–**7.2)**	**.027**		**4.4(1.4–13.4)**	**.008**
VPA (Middle TERTILE)	2.5 (0.9–6.4)	>.05		2.9 (0.9–8.8)	>.05
VPA (High TERTILE)—REF					

TPA: Total Physical Activity; MPA: Moderate Physical Activity; VPA: Vigorous Physical Activity

*Adjusted for Birthweight. BMI 1 year. BMI 2 years. Gestacional Weight Gain. Maternal Smoking during pregnancy. Scream and Sleep Time and Mother BMI and Education.
